# Occult Thyroid Carcinoma Localized to Three Cervical Lymph Nodes Without Primary Origin in the Thyroid Gland: A Case Report

**DOI:** 10.1155/crie/3603326

**Published:** 2026-02-23

**Authors:** Daisuke Murayama, Yasunori Nishida, Shun Hishikawa, Ryosuke Hirano, Toko Hashizume, Koji Azuhata, Hisashi Shimojo, Nobuo Ito, Osamu Mishima

**Affiliations:** ^1^ Department of Breast and Thyroid Surgery, Aizawa Hospital, Matsumoto, Nagano, Japan, ai-hosp.or.jp; ^2^ Department of Pathology, Aizawa Hospital, Matsumoto, Nagano, Japan, ai-hosp.or.jp

**Keywords:** cervical lymph node metastasis, neck lymph node metastasis, occult thyroid carcinoma

## Abstract

Occult thyroid carcinoma (OTC) involves cervical lymph node metastases without primary papillary thyroid carcinoma (PTC). A 57‐year‐old man presented with a left neck mass. Ultrasonography (US) revealed a mass in the left cervical node (inferior internal jugular node, Level IV). Positron emission tomography/computed tomography (PET/CT) and fine needle aspiration biopsy indicated suspected PTC metastasis. The serum thyroglobulin (Tg) level was elevated. Total thyroidectomy and bilateral central neck and left cervical dissection revealed no thyroid carcinoma, while the left cervical node showed ground glass nuclei and proliferation. Our diagnosis was PTC with no primary origin, pT0N1bM0 Stage II. Due to a low risk of recurrence, we initiated thyroid‐stimulating hormone (TSH) suppression therapy. Tg levels remained low 4 years postoperatively with no signs of recurrence. The optimal management of OTC cases remains unclear and blood tests (Tg levels), risk of recurrence, and patient characteristics must be considered.

## 1. Introduction

Papillary thyroid carcinoma (PTC) is often suspected from the presence of a cervical swelling and is currently diagnosed using screening techniques such as ultrasonography (US) or computed tomography (CT). Often accompanied by cervical lymph node metastases that are larger than the primary lesion in the thyroid gland, it is very rare that cervical lymph node metastases alone has been detected without a primary lesion in the thyroid gland, a condition termed “occult thyroid carcinoma” (OTC). Therefore, optimal diagnosis and treatment are difficult to achieve. Herein, we report a case of OTC localized to solely three cervical lymph nodes without primary origin in the thyroid gland.

## 2. Case Description

A 57‐year‐old man presented with a mass in the left side of the neck and had a medical history of hypertension. Physical examination revealed an elastic, mobile approximately 4 cm lesion, while US revealed a distinct mass in the left cervical node(inferior internal jugular node, Level IV) [1, 2] and a solid tumor in the right lobe of the thyroid (Figure 1). Fine‐needle aspiration (FNA) biopsy was performed on both lesions: the solid tumor in the right lobe of the thyroid was diagnosed to be benign and the mass in the left neck lymph node was suspected of PTC metastases. A CT scan showed a less‐enhanced mass in the left cervical node with no other lesions (Figure 2). Positron emission tomography (PET)/CT revealed strong mass‐like accumulation with a maximum standard unit value of 16.8, consistent with the enlarged left cervical node. However, no accumulation was observed in the thyroid gland (Figure 2). The thyroglobulin (Tg) level was 882 ng/mL (normal value: ≤33.7). Although the left cervical mass was not definitively diagnosed as malignant by FNA, it was strongly suspected to be a metastatic lymph node of an unidentified extremely micro‐PTC or PTC originating from the ectopic thyroid tissue. For postoperative radioactive iodine (RAI) therapy, total thyroidectomy and bilateral central neck and left cervical dissections were performed. Histological examination revealed no primary cancerous lesions in the thyroid gland. Cells with intranuclear cytoplasmic inclusions or nuclei grooves or ground glass nuclei were found in the left internal jugular node (Level IV) implying papillary‐like proliferation (Figure 3). TTF‐1 was positive in tumor cells. Tg, PAX8, BRAF, and cancer genome profiling tests were not performed. There were only three of 50 metastatic lymph nodes. We diagnosed OTC, pT0N1bM0 Stage II (Union for International Cancer Control). The solid tumor at the right lobe of the thyroid was diagnosed as adenomatous goiter. Although the lymph node was enlarged to a diameter of >3 cm, solely three lymph node metastases were localized in the left cervical node. Since the postoperative Tg level was extremely low (0.19 ng/mL), PTC originating from the ectopic thyroid tissue was ruled out and the risk of recurrence was considered low; therefore, we followed up with thyroid‐stimulating hormone (TSH) suppression. Four years postoperatively, the Tg level remained extremely low (0.16 ng/mL), with no signs of recurrence.

Figure 1Ultrasonography findings. (A, C) The right lobe of the thyroid, horizontal and sagittal images are shown, respectively. (B, D) The left inferior internal jugular node (Level IV), horizontal and sagittal images are shown, respectively. Arrow: a solid tumor in the right lobe of the thyroid 15.5 mm × 11.7 mm; asterisk: a distinct mass in the left neck lymph node 69.7 mm × 29.1mm. CA, carotid artery; IJV, internal jugular vein; SCM, sternocleidomastoid muscle; T, trachea.

**Figure:**
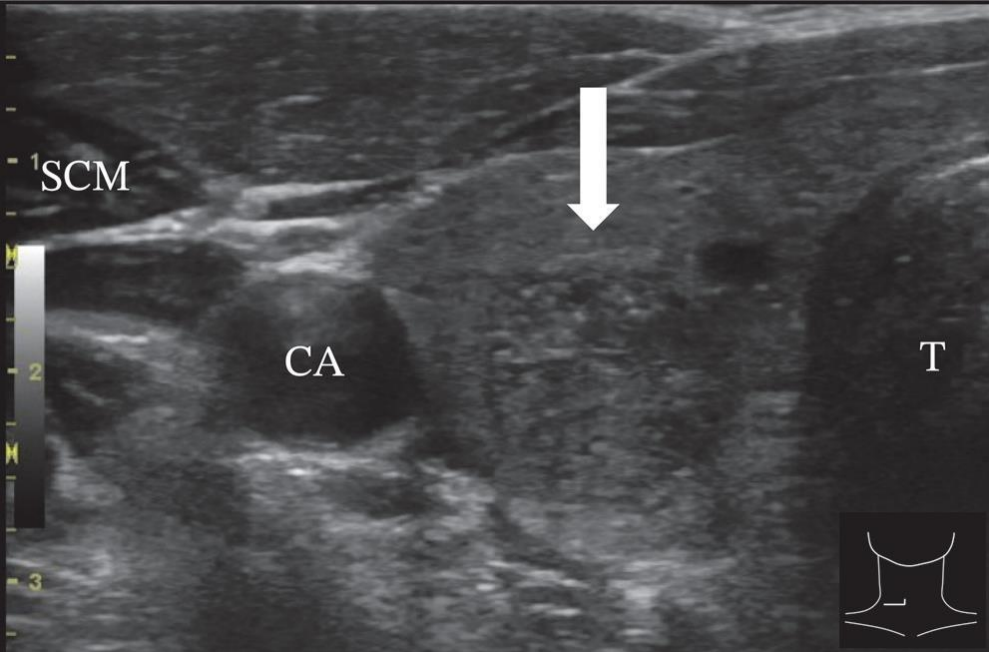
(A)

**Figure:**
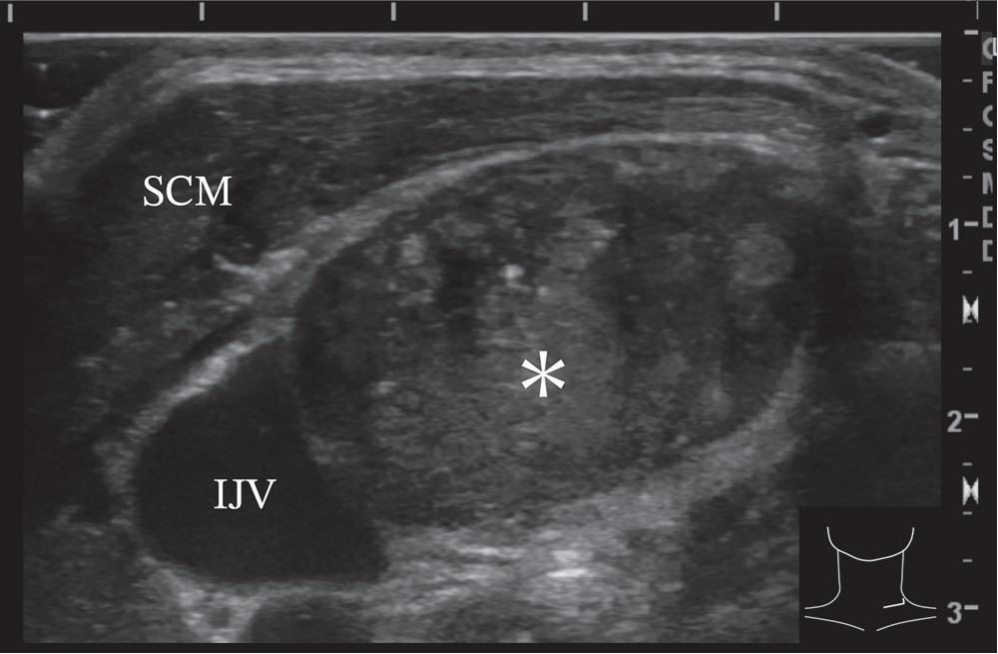
(B)

**Figure:**
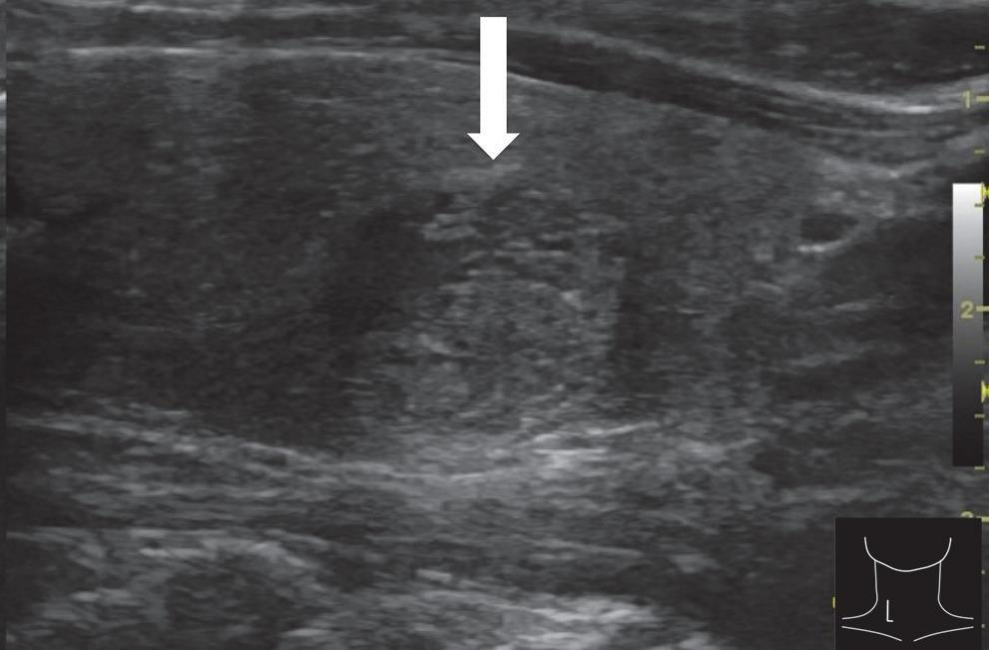
(C)

**Figure:**
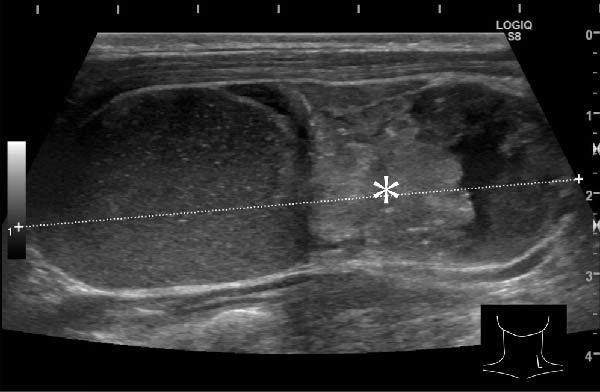
(D)

Figure 2Radiographs resulting from computed tomography (CT) and positron emission tomography (PET)/CT scans are shown. (A, C) The right lobe of the thyroid, CT and PET/CT scan images are shown, respectively. PET/CT scan revealed no accumulation in the tumor in the thyroid gland. (B, D) The left internal jugular node (Level IV), CT scan and PET/CT scan images are shown, respectively. PET/CT scan showed strong mass‐like accumulations with a maximum standard unit value of 16.8, consistent with the enlarged left cervical lymph node. Arrow: a solid tumor in the right lobe of the thyroid; asterisk: a distinct mass in the left neck lymph node.

**Figure:**
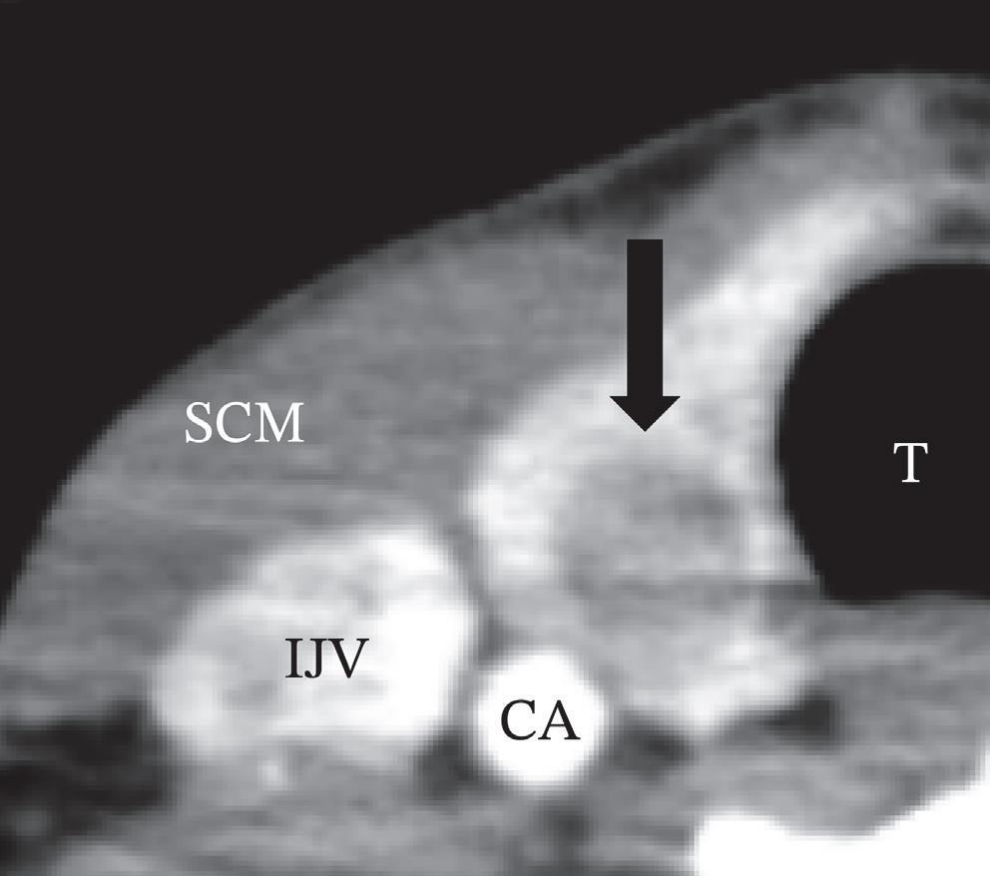
(A)

**Figure:**
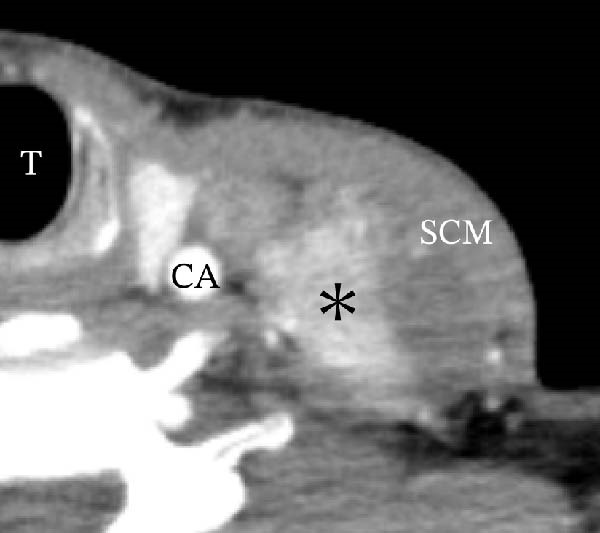
(B)

**Figure:**
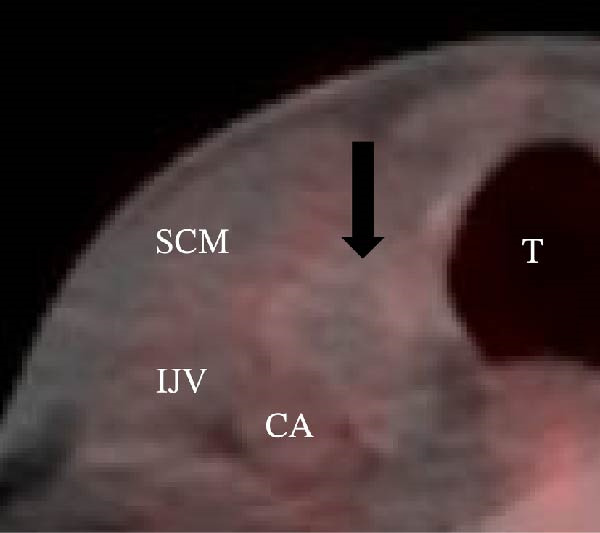
(C)

**Figure:**
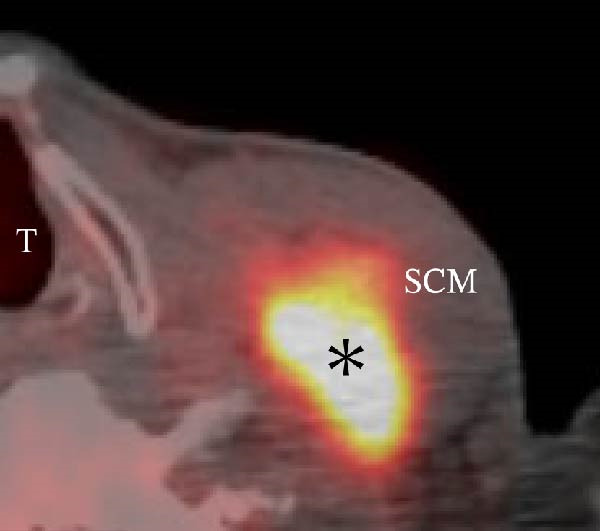
(D)

Figure 3Pathological findings. (A) Thyroid tissue: There was no primary cancerous lesion. (B) The left internal jugular node (Level IV). (C) Histological findings (hematoxylin and eosin staining): Cells with intranuclear cytoplasmic inclusions or nuclei grooves or ground glass nuclei were found, thereby showing papillary‐like proliferation.

**Figure:**
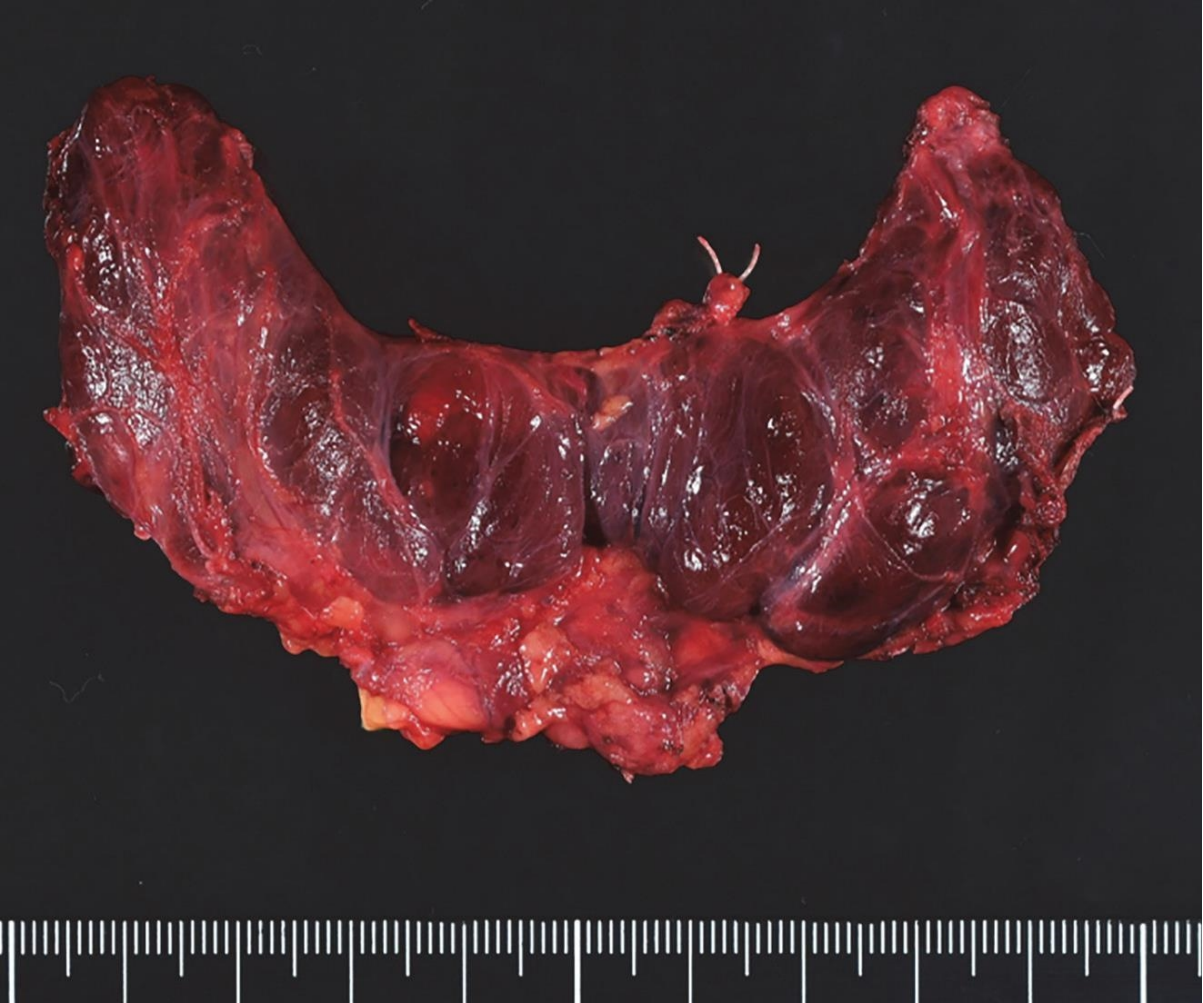
(A)

**Figure:**
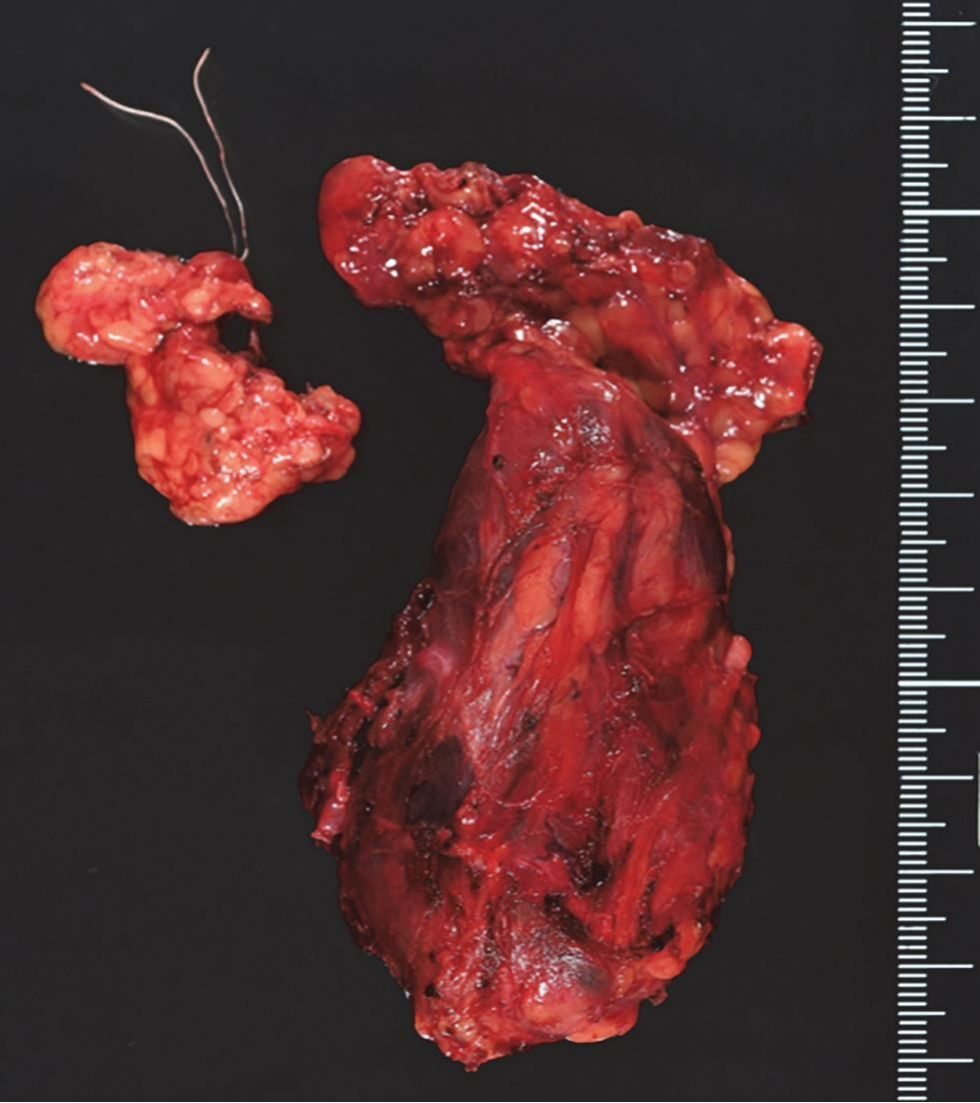
(B)

**Figure:**
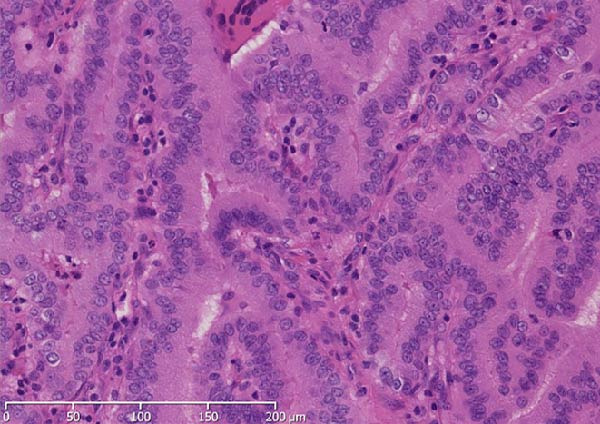
(C)

## 3. Discussion

Conventionally, OTC is defined as a <1.0 cm diameter impalpable thyroid carcinoma [3]. OTC is classified into five types, which are as follows: Type 1 is occasionally found on autopsy or surgery of a benign lesion (latent cancer) [4]. Type 2 is a micro‐PTC detected incidentally, mainly via US [4]. Type 3 is clinically apparent metastases from thyroid cancer, where the primary cancer is detected histologically [4]. Type 4 originates from an ectopic thyroid gland [4]. Type 5 is cervical lymph node metastasis of PTC without a primary origin in the thyroid gland (cancer of unknown primary origin) [5]. Currently, OTC is defined as PTC with clinically apparent node metastasis showing microscopic primary lesions overlooked by US [6]. Ito et al. [6] have reported that OTC was detected in only 17 (0.3%) of 5400 cases and in five (0.1%) patients, histology did not reveal thyroid gland cancer. Twelve patients (71%) had a single lymph node metastasis and the size of the metastatic lymph nodes was >3 cm in 12 of the 17 patients (71%) [6]. Most OTC cases have large (>3 cm) and localized lymph node metastases, which seems to apply to our case.

There are three possible hypotheses to explain why PTC can metastasize without any primary origin in the thyroid gland. First, <3 mm diameter microlesions may have missed detection [7]. Second, the primary tumor may have spontaneously regressed and disappeared. Regarding the mechanism of spontaneous tumor regression, Nishikawa et al. [8] revealed that diffusely infiltrated lymphocytes and macrophages associated with Hashimoto’s thyroiditis may play an important role. Spontaneous tumor regression is commonly observed in other carcinomas, such as hepatocellular carcinoma [9] and renal cell carcinoma [10], and is especially frequent in melanoma. Ribero et al. [11] reported that 10%–35% of melanomas regress spontaneously. Further, the histologic features of melanoma regression include fibrosis, which is often associated with lymphocytes or macrophages [12]. In our patient, since no macrophages or fibrotic changes were detected in the thyroid gland, spontaneous regression was considered improbable. Third, ectopic thyroid gland with benign lateral neck have been reported [13], moreover, primary thyroid carcinoma rarely occurs in these tissues [14]. Recently, in OTC case presenting with lymph node metastasis alone, Yamashita, et al. [15] have ruled out thyroid carcinoma originating from ectopic thyroid tissue within the lymph node, due to the absence of normal thyroid tissue within the metastatic lymph nodes. Also, in our case, no benign thyroid tissue was found in the lymph node metastasis. Also, in our case, no normal thyroid tissue was found in the lymph node metastasis.

In terms of surgical procedure, the National Comprehensive Cancer Network (NCCN) guidelines recommend total thyroidectomy and RAI for thyroid carcinoma with cervical lymph node metastasis. On the other hand, there is no consensus for managing OTC. In our OTC case, solely three lymph node metastases were localized, and postoperative Tg level was extremely low. Therefore, RAI was omitted because the recurrence risk was considered quite low. In accordance with the NCCN guidelines, we maintained TSH levels at 0.1–0.5 mU/L [16]. Postoperative management of OTC remained controversial.

In the study about the OTC cases from pathology database of Memorial Sloan Kettering Cancer Center from 2000 to 2016, although most OTCs were low grade and well differentiated, poorly differentiated or anaplastic carcinoma was also seen. Two‐third of the cases are BRAF V600E positive and all reported cases had papillary carcinoma phenotype [17].

As a limitation of our report, immunohistochemical studies except for TTF‐1 were not performed (Tg, PAX8, BRAF, and cytokeratin panels.). In addition, RNA–based comprehensive genomic profiling was also reported to be capable of detecting rare genetic mutations [18]. It might be valuable to perform these examinations in OTC for exclusion of malignant tumors other than thyroid carcinoma. Although RAI was omitted because the recurrence risk was considered quite low, functional imaging such as Tc‐99m scintigraphy or embryologic remnant assessment would be useful for diagnosis of OTC in preoperative phase. Future studies incorporating genomic profiling and systematic evaluation of ectopic thyroid remnants are warranted to clarify the pathogenesis and optimize management of OTC.

In conclusion, the risk of recurrence in our patient was estimated quite low, resulting in postoperative follow‐up with TSH suppression therapy alone. Since only a few OTC cases have been reported, the optimal management of OTC remains unclear. Nonetheless, total thyroidectomy and lymph node dissection, followed by RAI, should be performed. Blood tests (Tg levels), risk of recurrence, and patient characteristics must be considered.

## Funding

This research did not receive any specific grants from funding agencies in the public, commercial, or not‐for‐profit sectors.

## Disclosure

All authors have read and approved the final version of the manuscript. Corresponding author had full access to all of the data in this study and takes complete responsibility for the integrity of the data and the accuracy of the data analysis.

## Consent

Written informed consent for case publication was obtained from the patient.

## Conflicts of Interest

The authors declare no conflicts of interest.

## Data Availability

The datasets used in this study are available from the corresponding author upon request.
